# Hospital-based first diagnoses of systemic sclerosis: Defining initial clinical presentations and diagnostic pathways

**DOI:** 10.1515/rir-2026-0007

**Published:** 2026-03-30

**Authors:** Teerapat Luengthanapol, Tippawan Onchan, Patnarin Pongkulkiat, Ajanee Mahakkanukrauh, Siraphop Suwannaroj, Chingching Foocharoen

**Affiliations:** Department of Medicine, Faculty of Medicine, Khon Kaen University, Khon Kaen 40002, Thailand

**Keywords:** systemic sclerosis, scleroderma and related disorders, hospitalization, admission, diagnosis, community, social, pericardial effusion

## Abstract

**Background and Objective:**

Early diagnosis of systemic sclerosis (SSc) is challenging due to heterogeneous presentation and lack of reliable biomarkers, resulting in many patients being diagnosed during hospitalization with advanced disease and severe complications. We aimed to characterize the clinical presentation, diagnostic pathway, and outcomes of patients first diagnosed with SSc during hospitalization.

**Methods:**

We retrospectively reviewed adult patients newly diagnosed with SSc during hospitalization (January 2016-July 2023), including those referred for SSc evaluation or with SSc features identified during admission for other diagnoses. Patients previously diagnosed as outpatients were excluded. Clinical presentations were compared between patients admitted for SSc-related reasons *vs*. those admitted for other reasons subsequently diagnosed with SSc.

**Results:**

Thirty-five patients were included (mean age 60.4 ± 12.8 years; 60% women). Most (68.6%) were incidentally diagnosed with SSc during hospitalization for unrelated conditions. Common indications for hospitalization included pericardial effusion (22.9%), progressive pulmonary hypertension (17.1%), and progressive interstitial lung disease (14.3%). Approximately 60% of both hospitalized patients in both groups initially saw general practitioners before were rheumatological referral. Age, sex, symptom duration, most clinical features, length of stay, hospital costs, and outcomes were similar between patients admitted for SSC-related *vs*. non-SSc-related reasons, except for greater gastrointestinal involvement in the latter group (*P* = 0.02).

**Conclusion:**

Hospitalized patients first diagnosed with SSc predominantly present with diffuse cutaneous involvement and advanced cardiopulmonary complications. Most cases are identified during hospitalization for unrelated conditions, highlighting the need for increased clinical vigilance and systematic SSc evaluation in at-risk hospitalized patients to enable early detection and improve outcomes.

## Introduction

Systemic sclerosis (SSc) is a rare autoimmune rheumatic disease and connective tissue disorder characterized by fibrosis in multiple systems, including the skin, blood vessels, and internal organs.^[1]^ Although SSc has a relatively low prevalence—estimated at 50 to 300 cases per million globally and 240 cases per million in Thailand^[2]^–its burden at both the individual and healthcare system levels is substantial. The clinical progression and long-term outcomes of patients with SSc vary considerably. Among autoimmune rheumatic disorders, SSc is notable for its particularly high rates of disease-specific mortality and serious complications.^[3]^ SSc can lead to a multitude of serious complications, including interstitial lung disease (ILD), pulmonary hypertension (PHT), renal crisis, and significant gastrointestinal involvement, all of which contribute to increased morbidity, frequent hospitalizations, and substantial healthcare costs.^[4,5]^ The unpredictable and rapidly progressive nature of SSc poses considerable challenges for its early detection and management.

Scleroderma can be categorized into localized and systemic forms; SSc is further subclassified into limited (lcSSc) and diffuse cutaneous (dcSSc) subset. It can present with various clinical manifestations, particularly early signs, such as swollen and puffy fingers, musculoskeletal inflammation, general symptoms, scleroderma renal crisis, PHT, and Raynaud’s phenomenon.^[2,3]^ Detecting very early SSc can be challenging, as it may manifest as Raynaud’s phenomenon, puffy fingers, specific autoantibodies, and microvascular changes observed in capillaroscopy, while reliable biomarkers for diagnosis are currently lacking.^[6]^ Despite the improved sensitivity of the 2013 American College of Rheumatology (ACR) /European League Against Rheumatism (EULAR) classification criteria compared to the 1980 ACR criteria,^[7]^ there are still patients who display early signs of SSc but do not meet the 2013 ACR/EULAR Classification Criteria for SSc.^[8]^

SSc is the second most common reason for hospitalization among systemic connective tissue diseases, following systemic lupus erythematosus (SLE).^[4]^ Infection-related diseases account for the majority (60%) of hospitalizations in SSc patients, with pneumonia, cardiac issues, peripheral vascular disease, and PHT being the most common causes.^[5,9]^ Several factors, such as sex, race, digital ulcers, cardiac dysfunction, ILD, and PHT, are associated with both acute hospitalization and mortality.^[10,11]^

However, it has been observed that patients who are hospitalized and subsequently diagnosed with SSc usually present with advanced clinical features and complications of the disease. Late referral cases often involve cardiopulmonary issues and joint contractures, which are associated with high mortality, particularly due to ILD. Currently, no data is available specifying the common presenting symptoms of non-diagnosed SSc patients upon hospital admission. Therefore, further studies are needed to identify these symptoms and facilitate the early diagnosis and treatment of SSc.

Our objective was to define the initial clinical characteristics, diagnostic pathways, and outcomes of patients who were first diagnosed with SSc during hospitalization at a tertiary care center in Thailand. This study may help improve the early recognition and diagnosis of SSc. Understanding these early presentations and diagnostic processes will enable clinicians to identify the disease more promptly, which may lead to earlier intervention and improved patient outcomes. Additionally, insights gained from this study could inform the development of local guidelines and raise awareness among healthcare professionals, ultimately contributing to improved disease management in similar settings.

## Method

We conducted a retrospective study of adult patients diagnosed with SSc during hospitalization between January 2016 and July 2023 at Srinagarind Hospital, a university hospital in Khon Kaen, Thailand. Patients were included if they were referred specifically for SSc evaluation or if SSc features were incidentally identified during admission for other reasons. Patients diagnosed with SSc at the Out-patient Unit of Srinagarind Hospital before hospitalization were excluded. Data from electronic medical records and hard copies of medical records for the target population were reviewed and extracted from the Information Technology Division and the Health Information Management Department, respectively. The diagnoses were reviewed and verified by a rheumatologist before the data were included in the study. The clinical presentations of patients diagnosed with SSc during hospitalization were reviewed. The data collection included patient demographics, indication of hospitalization, primary diagnosis during hospitalization, clinical features of SSc and their onset, laboratory data, findings from chest radiographic study, high-resolution computed tomography (HRCT), echo-cardiogram, spirometry (if performed), serology results, procedure during hospitalization such as operation, right heart catheterization (if performed), length of stay, patient status, and causes of death (if died).

The patients hospitalized for the primary purpose of diagnosing SSc, with or without related complications. This group can be referred to as “SSc admission”. Patients hospitalized for other diagnoses who were subsequently diagnosed with SSc during their admission. This group can be referred to as “non-SSc admission”.

### Operational Definitions

A diagnosis of SSc is based on the 2013 ACR/EULAR Classification Criteria for SSc.^[7]^ SSc was classified as the lc-SSc or dcSSc subset as per LeRoy *et al*.^[12]^

The onset of the disease was defined as the appearance of the first non-Raynaud’s phenomenon symptom. The duration of symptoms before diagnosis was calculated as the interval between the date of diagnosis and the date of onset. Digital ulcers are painful, well-demarcated, denuded areas located on the volar surface of the fingers.^[13]^ Hand deformity refers to persistent flexion contractures of the finger joints, resulting in a claw-like appearance.^[14]^ ILD is diagnosed when interstitial fibrosis is detected on HRCT. Pulmonary arterial hypertension (PAH) is established when the mean pulmonary arterial pressure (mPAP) exceeds 20 mmHg at rest, coupled with a pulmonary artery wedge pressure of ≤ 15 mmHg and a pulmonary vascular resistance of ≥ 3 Wood units, as confirmed *via* right heart catheterization.^[15]^ PHT secondary to ILD is characterized by an mPAP between 20.0 and 34.9 mmHg along with a forced vital capacity below 70%, a forced expiratory volume in one second below 60% of the predicted value, or ≥ 20% parenchymal involvement as determined by HRCT.^[16]^ Esophageal involvement is present upon the manifestation of gastrointestinal symptoms associated with SSc, such as esophageal dysphagia, heartburn, or reflux.^[11]^ Stomach involvement is indicated by symptoms of early satiety or vomiting.^[17]^ Intestinal involvement is recognized by the presence of diarrhea, bloating, malabsorption, constipation, or evidence of ileus or pseudo-obstruction.^[11]^ Myocardial involvement is noted when the left ventricular ejection fraction is ≤ 50%.^[18]^ Scleroderma renal crisis is indicated by any of the following: a rapid and progressive increase in serum creatinine, sudden onset of hypertension, and/or microangiopathic hemolytic anemia.^[19]^ Musculoskeletal disorders were defined as the presence of myositis (inflammatory myopathy with proximal muscle weakness and elevated CK), tendon friction rubs, or inflammatory arthritis.^[20]^ Hypertensive urgency and emergency were defined by systolic BP ≥ 180 mmHg and/or diastolic BP ≥120 mmHg without and with acute target organ damage, respectively.^[21]^ Motility disorder was defined by the presence of gastrointestinal symptoms suggestive of neuromuscular dysmotility, including dysphagia, early satiety, bloating, diarrhea, or constipation, in the absence of mechanical obstruction.^[22]^

### Statistical Analysis

Descriptive data were reported as proportions or percentages for categorical variables and as means with standard deviations (SD) or medians with interquartile ranges (IQR) for continuous variables. A clinical comparison was performed between patients in SSc admission and non-SSc admission. Categorical data were evaluated using the *chi-square* test or Fisher’s exact test, while continuous data were analyzed using the Student’s *t*-test or Mann-Whitney U-test, as appropriate. The hospitalized mortality rate was calculated with a 95% confidence interval (95% CI). Statistical significance was set at *P* < 0.05. All statistical analyses were performed using STATA version 16.0.(StataCorp, College Station, TX, USA).

## Results

Thirty-five hospitalized patients with SSc were included in the review. The mean age was 60.4 years (SD 12.8), with 60.0% of participants being female. Most patients (24 cases, 68.6%) were non-SSc admission. dcSSc was the most common subset (74.3%), followed by lcSSc (17.1%). The primary pathological involvement was in SSc-related organ involvement (29 cases, 82.9%). The common indications for hospitalization included pericardial effusion (8 cases, 22.9%), progressive PHT (6 cases, 17.1%), and progressive ILD (5 cases, 14.3%). Of the eight patients with pericardial effusion, five (75%) had massive effusion and underwent pericardial window placement. Regarding the clinical characteristics of SSc, skin thickness was the most common manifestation (20 cases; 57.1%), while ILD (8 cases; 22.9%) and PHT (7 cases; 20.0%) were the main cardiopulmonary complications. Serologically, 57.1% of the patients were positive for anti-topoisomerase I antibody. Patient demographic data are shown in Table 1.

**Table 1:** Demographic data

Data	*N* = 35
Age (years), mean (SD)	60.4 (12.8)
Female, *n* (%)	21 (60.0)
Conditions of SSc during hospitalization, *n* (%)	
Hospitalization due to first diagnosis of SSc with and without its complications	11 (31.4)
Hospitalization due to other diagnosis and were diagnosed with SSc thereafter	24 (68.6)
SSc subsets, *n* (%)	
Limited cutaneous SSc	6 (17.1)
Diffuse cutaneous SSc	26 (74.3)
Overlap syndrome	3 (8.6)
Primary diagnosis categorized by pathological involvement	
SSc related	29 (82.9)
Non-SSc related	6 (17.1)
Indication of hospitalization*	
Pulmonary problems, *n* (%)	6 (17.1)
Progressive ILD	5
Pneumothorax	1
Cardiovascular problems, *n* (%)	17 (48.6)
Progressive PHT	6
Pericardial effusion	8
Left-sided heart failure	2
Right-sided heart failure	1
Renal problems, *n* (%)	5 (14.3)
Renal crisis	3
Non-SSC related AKI	2
Gastrointestinal problems, *n* (%)	1 (2.9)
MSK disorder, *n* (%)	2 (5.7)
Infection, *n* (%)	4 (11.4)
Pneumonia	2
Other infection	2
Clinical characteristics of SSc	
Skin involvement, *n* (%)	
Skin thickness	20 (57.1)
Salt and pepper skin appearance	3 (8.5)
Edematous skin	3 (8.5)
Vascular involvement, *n* (%)	
Digital ulcer	5 (14.3)
Raynaud’s	4 (11.4)
Telangiectasia	3 (8.6)
Digital gangrene	1 (2.9)
Musculoskeletal involvement, *n* (%)	
Arthritis	4 (11.4)
Myositis	2 (5.7)
Tendon friction rub	1 (2.9)
Gastrointestinal involvement, *n* (%)	
Esophageal reflux	5 (14.3)
Motility disorder	2 (5.7)
Cardiopulmonary involvement, *n* (%)	
ILD	8 (22.9)
PHT	7 (20)
Serological positive, *n* (%)	
Antinuclear antibody	27 (77.1)
Anti-topoisomerase I antibody	20 (57.1)
Anti-centromere antibody	8 (22.9)
Rheumatoid factor	1 (2.9)

* One patient had left-sided heart failure and pneumonia; one had progressive ILD and pericardial effusion; one had pericardial effusion and renal crisis; and one had pericardial effusion, renal crisis, left-sided heart failure, and pleural effusion. SD, standard deviation; SSc, systemic sclerosis; mRSS, modified Rodnan skin score; ILD, interstitial lung disease; PHT, pulmonary hypertension; IQR, interquartile range.

Clinical characteristics between SSc admission and non-SSc admission revealed no significant differences in age, symptom duration, or sex between groups. dcSSc was the predominant subset in both groups (90.9% and 66.7%, respectively). The proportion of dcSSc in the non-SSc admission group was higher than that of SSc admission; however, no statistical confirmation was obtained.

Progressive ILD (27.3% *vs*. 8.3%) and pericardial effusion (36.4% *vs*. 16.7%) were more common reasons for hospitalization in the SSc admission group than in the non-SSc admission group, although the difference was not statistically significant. Among the patients in non-SSc admission group, most cases of pericardial effusion were attributed to underlying SSc-related serositis or pulmonary hypertension. One patient had coexisting infectious pericarditis, while all others were consistent with SSc-related inflammatory effusion based on fluid analysis and clinical course. In contrast, progressive PHT, renal crisis, and musculoskeletal involvement tended to be more frequent in patients with non-SSc admission than in the other group; however, the difference was not significant. The only statistically significant difference between the groups was gastrointestinal involvement (9.1% *vs*. 16.7%, *P* = 0.02). Other clinical manifestations and serological findings were comparable between the two groups, with no significant differences observed (Table 2). Most SSc admission (63.6%) were seen by general practitioners and then referred to rheumatologists, while nearly 60% of patients with non-SSc admission were seen by general practitioners, cardiologists, pulmonologists, and nephrologists before being referred to rheumatologists. The length of stay for those hospitalized due to non-SSc-related reasons was slightly longer than that for those SSc admission, and hospital costs increased with longer stays, but the difference was not statistically significant (Table 2).

**Table 2:** Clinical comparison between SSc admission and non-SSc admission

Clinical data	SSc admission (*n* = 11)	Non-SSc admission (*n* = 24)	*P*-value
Age (years), mean (SD)	57.33 (12.45)	61.79 (13.04)	0.35
Duration of symptoms before diagnosis (months), median (IQR)	2.57 (1.03-10.2)	2.28 (0.57-13.33)	0.85
Female, *n* (%)	7 (63.6)	14 (58.3)	0.72
SSc subset, *n* (%)			0.49
Limited cutaneous SSc	1 (9.1)	5 (20.8)	
Diffuse cutaneous SSc	10 (90.9)	16 (66.7)	
Overlap syndrome	0 (0)	3 (12.5)	
Primary diagnosis categorized by pathological involvement, *n* (%)			0.28
SSc related	8 (72.7)	21 (87.5)	
Non-SSc related	3 (27.3)	3 (12.5)	
Indication of hospitalization			
Pulmonary problems, *n* (%)			0.28
Progressive ILD	3 (27.3)	2 (8.3)	
Pneumothorax	0 (0)	1 (4.2)	
Cardiovascular problems, *n* (%)			0.80
Progressive PHT	1 (9.1)	5 (20.8)	
Pericardial effusion	4 (36.4)	4 (16.7)	
Left-sided heart failure	0 (0)	2 (8.3)	
Right-sided heart failure	0 (0)	1 (4.2)	
Renal problems, *n* (%)			0.55
Renal crisis	0 (0)	3 (27.3)	
Non SSc related AKI	1 (9.1)	1 (4.2)	
Gastrointestinal problem	0 (0)	1 (4.2)	0.99
MSK disorder	0 (0)	2 (8.3)	0.99
Infection, *n* (%)			0.58
Pneumonia	0 (0)	2 (8.3)	
Other infection	2 (18.1)	0 (0)	
Clinical characteristics of SSc, *n* (%)			
Skin involvement	7 (63.6)	15 (62.5)	0.99
Vascular involvement	3 (27.3)	5 (20.8)	0.91
MSK involvement	2 (18.2)	4 (16.7)	0.17
GI involvement	1 (9.1)	4 (16.7)	0.02^*^
Cardiopulmonary involvement	4 (36.4)	6 (25)	0.64
Serology, *n* (%)			
Anti-centromere antibody	2 (18.2)	6 (25)	0.99
Anti-topoisomerase I antibody	6 (65.6)	14 (58.3)	0.28
First attending physician, *n* (%)			0.38
General practitioners	7 (63.6)	8 (33.3)	
Cardiologists	0 (0)	4 (16.7)	
Pulmonologists	0 (0)	1 (4.2)	
Nephrologists	1 (9.1)	1 (4.2)	
Rheumatologists	3 (27.3)	10 (41.7)	
Hospital outcomes			
Lengths of stay (days), median (IQR)	5 (3-14)	6.5 (3.5-13.5)	0.71
Hospital cost (THB), median (IQR)	21,759 (12,790.92-35,872.25)	39,550 (14,706.1-114,654.9)	0.23
Discharge status, *n* (%)			0.99
Improvement	11 (100)	22 (91.7)	
Death	0 (0)	2 (8.3)	

* Statistically significant. SSc, systemic sclerosis; SD, standard deviation; IQR, interquartile range; ILD, interstitial lung disease; PHT, pulmonary hypertension; MSK, musculoskeletal; GI, gastrointestinal.

All hospitalized patients with SSc admission improved and were discharged from the hospital, whereas two patients in the other group did not survive. One was a 75-year-old woman with dcSSc who presented with pericardial effusion; she died 10 days after admission due to heart failure. The other non-surviving patient was a 52-year-old man with SSc overlapping polymyositis, who died of sepsis due to a urinary tract infection 28 days after admission.

## Discussion

This retrospective study aimed to identify the clinical characteristics and initial presentations of patients diagnosed with SSc during hospitalization at a tertiary care center in Thailand. Most of our patients (68.6%) were first diagnosed with SSc during hospital admission for conditions unrelated to SSc rather than being hospitalized due to direct SSc complications. This finding aligns with prior studies suggesting that SSc, particularly in its early stages, is frequently under-recognized in outpatient and referral settings, resulting in diagnostic delays until more severe, hospital-requiring complications emerge.^[6,10]^

Although the proportion of patients who were SSc admission, was lower than that of non-SSc admission, there was a high proportion of dcSSc, significant cardiopulmonary involvement, and typical serological profiles among those SSc admission. This is consistent with the study by Distler *et al*., which showed that delayed presentation was frequently observed among patients with dcSSc, with many seeking care from their primary healthcare provider only after experiencing symptoms for as long as a year.^[23]^ A total of 84 physicians from six countries (the United Kingdom, France, Germany, Italy, Spain, and the United States) were interviewed in the study to assess how patients with dcSSc are referred, diagnosed, treated, and followed up in real-world clinical practice. The study also revealed that primary healthcare providers had different levels of awareness regarding the diagnosis of dcSSc. In some cases, the initial symptoms were not considered severe enough to prompt seeking medical care, leading to delays in referral and treatment. Our findings indicate that by the time patients were initially referred to our tertiary center and diagnosed with SSc, they were already in an advanced stage of the disease and required hospitalization. Improving early detection and referral processes could significantly reduce the risk of irreversible complications and long-term disability in these patients. Additionally, increased awareness among primary care professionals may facilitate faster diagnosis, improve patient outcomes, and ultimately reduce healthcare costs associated with managing advanced disease.

Our population had an average age of 60.4 years, with a female predominance (60%), consistent with global and Asia Pacific epidemiological data reporting a higher prevalence of SSc in women between the fourth and sixth decades of life.^[24,25]^ The high proportion of dcSSc (74.3%) surpasses the rates typically reported in Western cohorts (35–55%^[26, 27, 28, 29, 30, 31, 32]^ but mirrors data from other Asian studies,^[25]^ which have shown higher rates of dcSSc in hospital-based populations.^[5]^ In addition, there was a high frequency rate of anti-topoisomerase I (57.1%) antibody positivity, which is more likely to be present in the dcSSc subset, and a lower frequency of anti-centromere antibody (22.9%) in our hospitalized SSc population, fitting the latter’s association with lcSSc. The anti-topoisomerase I antibody is known to be associated with poor prognosis in SSc.^[33,34]^ The findings indicate that our hospital is a tertiary center, and there is a referral and selection bias towards more severe and complicated cases. dcSSc and positive for anti-topoisomerase I antibody, which are more aggressive and associated with earlier and more severe organ manifestations, predominated. This may reflect both referral bias and more aggressive disease in the Asian population.

Cardiac and pulmonary complications were frequent clinical features for hospitalization, comparable to previous studies.^[5,35]^ Of these, pericardial effusion was the most common indication for hospitalization in our study (22.9%). Pericardial effusion was reported in approximately 15% of patients with SSc with asymptomatic cardiac involvement on echocardiography;^[36,37]^ however, symptomatic pericarditis or cardiac tamponade was rare.^[38]^ Pericardial effusion was also rarely reported as the first presentation in SSc patients,^[39]^ and pericardial involvement requiring hospitalization is uncommon in this population.^[40,41]^ Once hospitalization occurs due to pericardial effusion, the effusion is likely to be large or clinically significant, and procedures, particularly pericardial window, and/or special treatments are needed to evaluate the nature of the disease and control it.^[41]^ In patients with SSc, a significant amount of pericardial effusion can sometimes serve as an initial clinical manifestation of underlying PHT.^[42]^ This may explain why our patients had a high proportion of both pericardial effusion and progressive PHT.

The predominance of circulatory and respiratory system involvement, as well as complications such as ILD and PHT, reflects the organ systems most likely to precipitate hospitalization, either due to rapid clinical decompensation or secondary complications such as renal impairment or heart failure. In contrast to outpatient cohorts, in which Raynaud’s phenomenon is typically the first symptom,^[6]^ many patients in this study did not report or have Raynaud’s documented at admission. Through medical record review, Raynaud’s phenomenon was recorded only if explicitly documented in the admission records or patient history, so it was likely underreported due to retrospective data limitations. Additionally, due to the acute complications in most hospitalized patients, vasculopathy may not have been a clinical focus. This likely reflects delayed recognition in the community and the fact that patients usually present for hospitalization only after developing advanced organ involvement. Moreover, vasculopathy generally follows a more indolent course, making such symptoms unlikely to lead to hospitalization.

For the primary diagnosis, respiratory symptoms were slightly more common, with more cases of progressive ILD (27.3%) leading to hospitalization in SSc admission group, than in those non-SSc admission group. Gastrointestinal (GI) involvement was significantly higher in non-SSc admission group (16.7% *vs*. 9.1%, *P* = 0.02). This may indicate that GI symptoms in SSc are often attributed to common conditions, such as gastroesophageal reflux disease (GERD), leading to delayed diagnosis.^[43]^ Renal crisis and pneumonia were more frequently observed among patients in non-SSc admission group, suggesting that SSc may remain unrecognized until complications arise.^[44]^ Musculoskeletal disorders and hypertensive crises were infrequent overall but were only seen in the non-SSc admission group, indicating that specific symptoms are more likely to be misclassified before SSc is diagnosed.^[45]^

Our findings highlight that hospitalized patients newly diagnosed with SSc most often present with dcSSc and advanced organ involvement, particularly affecting the heart and lungs. Clinicians in both medical and surgical inpatient settings should maintain a high index of suspicion for SSc, especially in patients with unexplained multisystem diseases (cardiac, renal, and respiratory) and compatible serological markers or skin findings. The predominance of anti-topoisomerase I antibody positivity supports its added value as a diagnostic tool in suspected cases of progressive lung or cardiac involvement.

Most patients consulted general practitioners or specialists other than rheumatologists according to their main clinical presentation, even when clinical features of established SSc were present. The initial physician plays a critical role, as diagnostic errors or poor clinical decisions can delay appropriate treatment and worsen patient outcomes. Based on our observations, patients hospitalized for another diagnosis and subsequently diagnosed with SSc were usually seen by general practitioners. Furthermore, the length of stay in non-SSc admission group was slightly longer than SSc admission group, and hospital costs increased with longer stays. These findings indicate that clinical presentations other than SSc itself leading to delays in assessment and treatment and/or poor outcomes.

Our study had several limitations. First, the retrospective design carries a risk of missing or incomplete data, particularly for features not well documented prior to admission. Many hospitalized patients with life-threatening visceral complications had incomplete skin thickness recorded, even though they met dcSSc classification criteria based on other features. Second, referral and selection biases likely skewed the cohort towards more severe or advanced SSc. Finally, the sample size (*n* = 35) was relatively small, which limited the statistical power for subgroup analyses, as is common for rare diseases. The study was single-center and hospital-based; thus, it is unlikely to be reflective of the broader SSc population in the community, especially patients with the lcSSc subset or milder manifestations who do not require hospitalization. Although our sample size was limited due to the rarity of first-time inpatient SSc diagnosis, our findings are consistent with other hospital-based cohorts reporting advanced disease at diagnosis. We have added a summary comparison of key studies to contextualize our results between the present study and the previous studies focusing on initial clinical presentations of SSc in Table 3. Future research should include pooled multicenter datasets or national registries to increase statistical power and improve generalizability.

**Table 3:** Summary of comparisons between the present study and the previous studies focusing on initial clinical presentations of SSc

Study (year)	Country	*N*	dcSSc	Major first complications	Notable findings
Our study (2025)	Thailand	35	74.3%	Pericardial effusion 22.9%, ILD 22.9%, PHT 20.0%	Most first diagnosed during admission for non-SSc reasons
Netwijitpan, *et al*.^[5]^ (2013)	Thailand	157	70.3%	Infection, PHT, ILD	ILD predicted mortality
Caetano *et al*.^[9]^ (2022)	Portugal	99	60.0%	ILD, heart failure, renal crisis	Infection leading cause of admission
Potera, *et al*.^[35]^ (2022)	US	701	–	Respiratory failure, PHT, heart failure	PHT strongest predictor of mortality
Distler, *et al*.^[23]^ (2018)	Multinational	376	66.0%	Not inpatient study	Delayed referral most common in dcSSc
Bellando-Randone, *et al*.^[6]^ (2019)	Italy	53 VEDOSS	0% (early disease)	Raynaud’s + ANA	Early detection reduces progression

dcSSc, diffuse cutaneous systemic sclerosis; PHT, pulmonary hypertension; ILD, interstitial lung disease; VEDOSS, very early diagnosis of systemic sclerosis.

The strengths of our study are its unique approach to addressing a critical knowledge gap by specifically investigating the initial clinical characteristics and outcomes of patients who received their first SSc diagnosis during hospitalization. By excluding patients already diagnosed as outpatients, the study maintained a focused cohort, which enhanced the precision of the findings related to the clinical presentation and outcomes of newly diagnosed in-hospital cases. These findings may help improve patient care and guide educational efforts targeting primary care and internal medicine providers.

**Figure 1:**
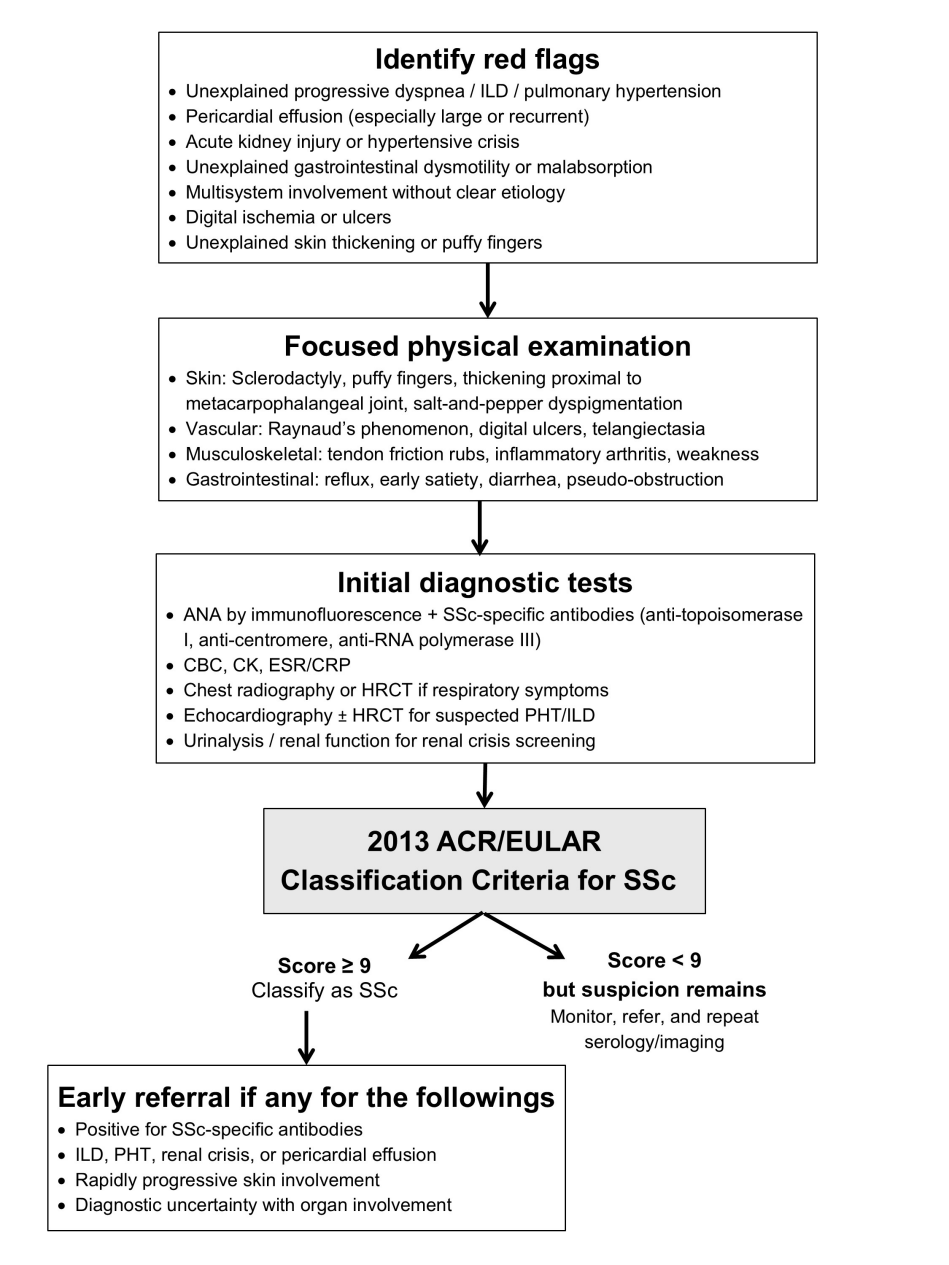
Proposed diagnostic pathway for hospitalized patients who are admitted with non-SSc diagnoses or unexplained multisystem involvement. ANA, anti-nuclear antibody; CK, creatine kinase; CRP, C-reactive protein; ESR, erythrocyte sedimentation rate; HRCT, high-resolution computed tomography; ILD, interstitial lung disease; PHT, pulmonary hypertension; SSc, systemic sclerosis.

We suggest that future studies include multicenter and prospective data to capture a broader spectrum of incidents of first diagnoses of SSc during hospitalization and examine how initial presentation and time to diagnosis impact outcomes in diverse populations. Studies assessing barriers to early diagnosis, the utility of emerging biomarkers or imaging, and the impact of educational interventions on prompt referral and diagnosis are needed. Finally, studies comparing survival, long-term organ function, and health service utilization outcomes between patients diagnosed in hospitals and outpatient settings could further clarify optimal care pathways. Based on our study objectives and findings, we propose a diagnostic pathway for hospitalized patients admitted with non-SSc diagnoses or unexplained multisystem involvement, as illustrated in Figure 1.

## Conclusion

For many patients in our tertiary center, SSc was first recognized during hospitalization, often at a stage of advanced organ involvement. These observations highlight the need for improved awareness and earlier identification of SSc in outpatient and primary care settings. Most of these patients already had advanced disease and serious organ involvement at the time of admission, especially dcSSc, ILD, and cardiopulmonary complications, regardless of whether the hospitalization was due to the initial SSc diagnosis or another reason, with SSc diagnosed subsequently. These findings underscore the importance of heightened awareness and close clinical monitoring for the early detection of SSc, particularly in patients with unexplained multisystem involvement. Early recognition and referral strategies, as well as improved diagnostic resources at the primary care level, are crucial for preventing disease progression and reducing the hospitalization rates. Further large-scale, prospective, multicenter studies are necessary to confirm these patterns and develop interventions aimed at promoting early diagnosis and improving outcomes for patients with SSc in both hospital and community settings.
